# Nanoparticle-Encapsulated Liushenwan Could Treat Nanodiethylnitrosamine-Induced Liver Cancer in Mice by Interfering With Multiple Critical Factors for the Tumor Microenvironment

**DOI:** 10.3389/fphar.2020.01052

**Published:** 2020-07-10

**Authors:** Jing Hong, Xi-Zhen Chen, You-Gong Peng, Wei Kevin Zhang, He-Bin Tang, Yu-Sang Li

**Affiliations:** ^1^Laboratory of Hepatopharmacology and Ethnopharmacology, School of Pharmaceutical Sciences, South-Central University for Nationalities, Wuhan, China; ^2^Department of General Surgery, The Second People’s Hospital of Jingmen, Jingmen, China

**Keywords:** liver cancer, nano-LSW-low, low toxicity, tumor microenvironment, nanodiethylnitrosamine

## Abstract

We previously isolated an ethanol fraction of LSW (Liushenwan pill, a traditional Chinese medicine) which has been shown to prevent and treat liver cancer induced by nanodiethylnitrosamine (nanoDEN) in mice. In the present study, we utilized a high-pressure microfluidics technique to generate LSW lipid nanoparticles (nano-LSW) to reduce its toxicity, and enhance its inhibitory effect on tumor growth, and further evaluate its therapeutic effect using a nanoDEN-induced mouse model of liver cancer. Our *in vitro* results indicated that nano-LSW-low could induce apoptosis in HepG2 cells, but exhibited low toxicity in L02 cells. Furthermore, the *in vivo* results indicated that nano-LSW-low exerted minimal or no damage to normal hepatocytes, kidney, and small intestine tissues. In addition, our results showed that at the 20^th^ week, the inflammatory infiltration in the mice in the model group increased severely, and partial pimelosis and fibrosis occurred. In contrast, the liver tissues in the mice treated with nano-LSW exhibited only slight inflammatory infiltration, without pimelosis and fibrosis. At the 30^th^ week, 4 out of 5 liver tissues in the model group showed hyperplastic nodules by hematoxylin and eosin (H&E) staining. However, the liver tissues in the nano-LSW treatment group did not showed hyperplastic nodules. Immunohistochemical staining showed that, in contrast to the model group, the levels of COX-2, PCNA, β-catenin, and HMGB1 protein expressions were significantly lower in the nano-LSW-low group at the 20^th^ and 30^th^ week. Compared to model group, the *COX-2*, *TNF-α*, *Smad-2*, and *TGF-β1* mRNA levels obviously decreased in the liver tissue after the nano-LSW-low treatment. Taken together, nano-LSW-low may serve as a potent therapeutic agent for preventing liver cancer by interfering with multiple critical factors for the tumor microenvironment during oncogenesis.

## Introduction

Liver cancer has become one of the leading causes of death among cancer patients worldwide (Siegel et al., 2014). Liver cancer ranks sixth in incidence and third mortality among malignant tumors, and approximately 500,000 new cases are diagnosed each year (Khalid and Bouneva, 2011; Valery et al., 2018). Unfortunately, many patients have advanced stage disease at the time of diagnosis. Therefore, the 5-year overall survival rate in patients is only 30%‑40% (Tai et al., 2017). Liver cancer develops in three stages: initiation, promotion and progression. During these three developmental stages, genetic and epigenetic changes sets the stage for progression toward inflammation, steatosis, fibrosis or cirrhosis, and eventually to liver cancer (Li et al., 2016b; Jiang et al., 2019). As a carcinogen, nanoDEN can induce these changes in the microenvironment to promote the development and progression of liver cancer (Zhang et al., 2017b).

The current treatment options for liver cancer may include surgery, chemotherapy, radiation, traditional Chinese medicine, or a combination of these therapies. Over the years, the research on the anti-cancer effects of traditional Chinese medicine has been very active and has made good progress (Zhang et al., 2018a). Liushenwan pills (LSW, a compound medicine) is one of the most famous Chinese patent medicines in China. LSW is made from six ingredients: musk (4.5 g), realgar (3 g), venenum bufonis (3 g), bezoar (4.5 g), borneol (3 g), and pearl (4.5 g) with a content of 3.125 grams per 1000 pills. It has effects of “heat clearing, detoxicating, and pain-relieving”. Meanwhile, recent studies have shown that LSW has anti-inflammatory and analgesic activities and good anti-cancer effect (Ma et al., 2007; Lin et al., 2014; Chen et al., 2018).

Global nanotechnology is rapidly developing. Nanomaterials exhibit improved bioavailability, solubility, and targeting as well as slower release properties than traditional drugs (Aires et al., 2016; Piktel et al., 2016). Therefore, nanomaterials exhibit improved the efficacy and reduced adverse reactions, which makes them widely used in the fields of drug delivery, gene therapy, and cancer treatment (Bhattacharyya et al., 2011; Zhao et al., 2015b). In our previous study, we observed a better anti-tumor effect of the alcohol extract supernatant of LSW (LSW-ET, concentration of 24.089 mg/ml; (Chen et al., 2018).

Therefore, in the present study, we used the lipid nano drug- system to chemically nanosize the LSW alcohol extract (LSW-ET), generated low- and high-loading nanoparticle-encapsulated LSW-ET, and evaluated its efficacy in preventing liver cancer induced by nanoDEN. Furthermore, we investigated the changes in the expression of multiple critical factors (including COX-2, TNF-α, β-catenin, HMGB1, PCNA, PPAR-γ, AP-2, Smad-2, TGF-β1) for the tumor microenvironment after treatment with nanoparticle-encapsulated LSW.

## Methods

### Materials

The following drugs were used: LSW (Lei Yun Shang Pharmaceutical Co., Ltd., Shanghai, China. batch number: 140701); diethylnitrosamine (DEN; Tokyo Chemical Industry Co., Led., Tokyo, Japan); sesame oil (Blessing Mill, Wuhan, China); lecithin from egg yolk (Cat. No. 6901933, Sinopharm Chemical Reagent Co., Ltd., Shanghai, China); Tween-80 (Cat. No. 0442) and glycerol reagent plus [Cat. No. G7757; Gaschromatography (GC) grade; Sigma-Aldrich Co., St Louis, MO, USA]; ultrapure deionized water (Heal Force Biomeditech Holdings Ltd., Shanghai, China). All the other reagents in the experiment were pure grade for analysis. The primary antibodies used in this experiment included antibodies against COX-2 (Cat. No. ab23672), β-catenin (Cat. No. ab6301), PCNA (Cat. No. ab29), and HMGB-1 (Cat. No. 18256; Abcam Inc., Cambridge, MA, USA). RNAiso Plus (Cat. No. H9108A) was provided by Takara Bio Group (Dalian, China).

### Animal Care

Male Kunming mice (18-22 g, 6 weeks of age) were provided from the Laboratory Animal Center, Hubei. The mice were housed for 7 days in specific pathogen free conditions with thermoregulatory environments (22–25°C) and a 12-h light-dark cycle, and had free access to water and food. In this study, all the experimental protocols were carried out according to the Committee of Research Facilities for Laboratory Animal Sciences and were approved by the Committee on the Ethics of Animal Experiments of the South-Central University for Nationalities, Wuhan, China (2012-SCUEC-AEC-002).

### Preparation of the Nanosized Materials

To prepare nanosized LSW-ET materials, a high-pressure microfluidics technique was used as follows. LSW-ET powder was dissolved in the oil phase (72% sesame oil, 6% egg yolk lecithin) and heated to 60°C. The oil phase was mixed with the liquid phase (1.25% Tween 80, 2.81% glycerol, 95.94% water) at a ratio of 1:4 at 60°C, followed by high speed shearing at 10,000 rpm for 5 min. Furthermore, the formed coarse emulsion was then homogenized by passing it through an Avestin high-pressure homogenizer EmulsiFlex-B15 (Ottawa, Canada) for 5 cycles at 1000 bars. After cooling down to room temperature, the dispersion was filter-sterilized (a 0.22 µm cellulose acetate filter; (Zhao et al., 2015a). The final concentrations were 24.089 mg/ml LSW in nano-LSW-high and 4.818 mg/ml LSW in nano-LSW-low. Nanovehicle was also prepared using the same procedure but lacking LSW-ET powder.

### Measurement of the Particle Size and Zeta Potential of the Nanosized Materials

We measured the particle size and potential using a Zetasizer nano ZS90 (Malvern Instruments, Malvern, UK). The nano-LSW-high and nano-LSW-low were diluted with ultrapure water until the count rate was between 200-300 Kcps (1000 counts per second) when we measured the particle size. To determine the zeta potential, the nano-LSW-high and nano-LSW-low were diluted with a 0.9% sodium chloride solution until the conductivity of the dilute suspension was 50 S/cm (Liang et al., 2019). The mean value of triplicates for each measurement is presented.

### Field Emission Scanning Electron Microscopy (FE-SEM)

The morphology of nano-LSW-high and nano-LSW-low was observed using a Hitachi SU8010 cold field emission scanning electron microscope (SEM; Tokyo, Japan). The newly prepared nano-LSW-high and nano-LSW-low were diluted 50 times with ultrapure water and sonicated for 1 min. A few drops of diluted nano-LSW-high (or nano-LSW-low) were placed on a carbon-coated copper grid (400 mesh; Beijing Xinxing Braim Technology Co., Ltd., Beijing, People’s Republic of China) and allowed to adsorb for 5 mins. Next, excess liquid was blotted with absorbing paper. After drying naturally for 1 h, the dried specimen was visualized using a SEM at an acceleration voltage of 35 kV (Havrdova et al., 2014).

### HPLC Analysis of Nano-LSW

LSW-ET is a crude extract, we detected the chemical components of nano-LSW using HPLC DionexTM Ultimate 3000 system (Thermo Fischer Scientific, Inc., USA). The separation of nano-LSW was carried out by a C18 column (4.6 × 250 mm, 5 μm; Kromasil). The mobile phase we have optimized consisted of acetonitrile (solvent A) and 0.2% formic acid water solution (solvent B, v/v), which flow rate was 0.8 ml/min. We used the following combination conditions for elution: 9% solvent A for 0‑3 min; 9%‑12% solvent A for 3‑13 min; 12%‑20% solvent A for 13‑15 min; 20%‑30% solvent A for 15‑25 min; 30%‑51% solvent A for 25‑30 min; 51% solvent A for 30‑60 min [total runtime 60 min; (Zhang et al., 2018b)]. The detection was performed at 296 nm and column temperature of 30°C.

### *In Vivo* Release Studies

In this study, we used the dialysis bag diffusion technique to evaluate the *in vitro* release of nano-LSW-high and nano-LSW-low. A total of 1 ml of nano-LSW-high and nano-LSW-low were placed in dialysis bags (MW: 14000). Then, 100 ml of PBS (0.01 M; pH 7.4) was added into the dialysis bags at 37°C in a QYC-200 shaking incubator at 100 rpm. Then, 2 ml of the released medium was withdrawn at 10 mins, and 0.5, 1, 2, 4, 8, 12, and 24 h, and replaced with 2 ml fresh PBS to maintain a constant volume. The release medium was extracted with 60 ml of methanol (extraction 3 times). Next, the extracts were combined, and their organic layer was transferred to a rotary evaporator and concentrated to 1 ml in a water bath at 50°C. Then, the 1 ml sample was analyzed by HPLC.

### Apoptosis Analysis

L02 (Cat. No. GDC079) and HepG2 (Cat. No. GDC141) cells were purchased from the Chinese Academy of Sciences (Shanghai, China). In the present study, the cell deaths of two types of cells were measured using an Annexin V-FITC/PI apoptosis detection kit. First, L02 and HepG2 cells (1×10^6^ cells/ml) were plated into 6-well plates in DMEM supplemented with 10% FBS, and then treated with LSW-ET (5 ul/ml, concentration of 120.445 µg/ml), nano-LSW-low (5 ul/ml, concentration of 20.09 µg/ml), or nano-LSW-high (5 ul/ml, concentration of 120.445 µg/ml) for 24 h, respectively. Then, cells were treated with Annexin V-FITC/PI dye according to the manufacturer’s instructions, and apoptosis was assessed using a Guava easyCyte 5 Flow Cytometer (Merck, Darmstadt, Germany).

### Acute Toxicity Study of Nano-LSW-Low

Forty mice were randomly and equally segregated into four groups (n=10). The LSW, LSW-ET and nano-LSW-low groups received LSW, LSW-ET, nano-LSW-low, respectively, *via* intragastric administration (4.818 mg/ml, 0.4 ml/10 g), one time for each group. The control group was given the same volume of physiological saline. Then, the liver, kidney and small intestine from the mice were collected for analyses of weight and histology on the 14^th^ day.

### *In Vivo* Experiments With the Synthesized Nanoparticles

One hundred and sixty young male Kunming mice were randomly divided into five groups: the control group, nano-DEN group, LSW-ET group, and nano-LSW-low group, nano-LSW-high group. All groups were orally administered nanoDEN (16.5 mg/kg) once a week for 20 consecutive weeks, except for the control group which was administered 0.9% saline. The LSW-ET (9.639 mg/kg in sesame oil) group was treated orally with LSW-ET. The nano-LSW-low group received oral nano-LSW-low (1.927 mg/kg, 4 ul/10 g). The nano-LSW-high group received orally nano-LSW-high (9.639 mg/kg, 4 ul/10 g). The three groups were fed twice a week until the 30^th^ week start of the experiment. Body weights were measured daily until the mice were sacrificed at week 10, 15, 20, and 30 (n=8, the first administration of nanoDEN was week 0). Liver samples from all mice were harvested for downstream analyses histology and mRNA expression analyses.

### Histopathological Analysis of the Liver Tissues

Fresh tissues harvested from the liver were immediately fixed in 10% neutral formalin, and embedded in paraffin. Then, 2 µm-thick sections were sliced, adhered to microscopic slides, and stained with H&E [(Li et al., 2016a). Histopathologic examinations of the liver sections were performed by a pathologist using a Nikon 50i light microscope (Nikon Inc., Tokyo, Japan].

### Immunohistochemical of the Liver Tissues

In brief, the paraffin-embedded liver tissue sections were deparaffinized and hydrated, followed by antigen was retrieved by citric acid buffer (pH = 6.0) microwave antigen retrieval, and the endogenous peroxidase was blocked with a 3% H_2_O_2_. Subsequently, the sections were blocked with 5% BSA (Cat. No. 36106ES25; Yeasen), then incubated with primary antibodies against PCNA (1:6000), COX-2 (1:200), β-catenin (1:200), HMGB-1 (1:1000), and secondary antibody, respectively. After color development with 3,3’-diaminobenzidine tetrahydrochloride, the sections were counterstained with hematoxylin and mounted using coverslips with aqueous mounting media. Finally, the spectral optical density of the stained sections was automatically acquired from 420 to 720 nm in 10 nm increments with a CRi Nuance Multispectral Imaging System (Cambridge Research and Instrumentation Inc., Woburn, MA, USA) as described in our previous study (Li et al., 2012).

Spectral unmixing for each image was performed using Nuance software (v3.0.2) and pure spectral libraries of individual chromogens. The DAB single-channel image and hematoxylin single-channel image are obtained by spectral decomposition. The total signal (optical density) of marker of co-localization in DAB image is the total expression of protein-positive regions. The total signals (optical density) of marker of co-localization in DAB image and hematoxylin image are the protein expression level located in the nucleus. The difference between the DAB channel and hematoxylin channel protein expression level is used as the cytoplasmic expression. The measured background noise was subtracted from each image. Three equal-sized fields were chosen at random to quantify the expression of the corresponding protein in each image.

### Quantitative Real-Time RT-PCR

Total RNA was harvested from the liver tissues with RNAiso Plus (Cat. No. H9108A, TaKaRa, Dalian, China), followed by cDNA synthesis with a PrimeScript II First Strand cDNA Synthesis kit (Cat. No. 6210A-1, TaKaRa) according to the manufacturer’s instructions. Quantitative real-time PCR was performed on a TP800 Thermal Cycler Dice System (TaKaRa Bio, Japan) using SYBR Premix Ex Taq II (Cat. No. RR820A, Takara) with 40 cycles of 95°C for 5 s and 60°C for 30 s. GAPDH was used as an internal standard. The primer pairs used in the present study are shown in **Supplementary Table 2**.

### Statistical Analysis

All data are expressed as the mean ± SEM. All statistical investigations of the differences among the groups were analyzed by one-way ANOVA, followed by Bonferroni’s *post hoc* tests. *P* < 0.05 was considered to be statistically significant, and the levels of differences were denoted in the caption of the corresponding figure. The statistical graph is drawn based on the relative value calculated by the protein expression of all groups in comparison with the average value of the protein expression of the control group. The statistical graph is drawn based on the percentage of the expression of each group of proteins in the average value of the expression of the control group. Compared with the control group, ∗, P<0.05; ∗∗, P<0.01; †, P > 0.01. Compared with the nanoDEN group, #, P<0.05; ##, P<0.01; ‡, P > 0.01.

## Results

### Characterization of Nano-LSW

**Figure 1:**
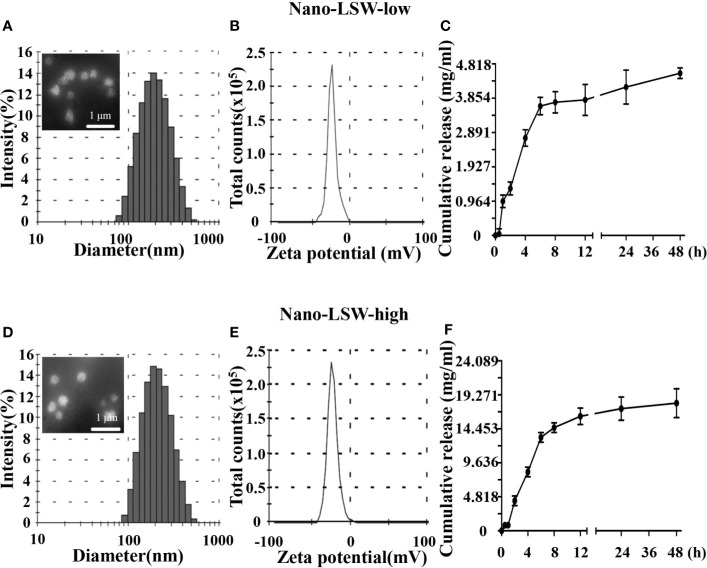
**(A)** Distribution of the diameters and the FE-SEM image in the top left corner, **(B)** distribution of the zeta potentials, and **(C)** the cumulative release profiles of the nano-LSW-low. **(D)** Distribution of the diameters, the FE-SEM image in the top left corner, **(E)** distribution of the zeta potentials, and **(F)** the cumulative release profiles of the nano-LSW-high.

The size distribution, zeta potential, morphology, and cumulative release of nano-LSW are shown in **Figure 1**. The average diameter of nano-LSW-low particles was ~201 nm, and the particles were round spheres in shape and had a smooth surface (**Figure 1A**). The zeta potential of nano-LSW-low was -23.6 mV ± 6.87 mV (**Figure 1B**). The *in vitro* release profile of nano-LSW-low showed that greater than 70% LSW was released in the first 6 h. At 48 h, 90% of LSW was released (**Figure 1C**). The size and zeta potential of nano-LSW-high were 201 nm and -26.3 mV ± 6.62 mV, respectively. The *in vitro* release profile of nano-LSW-high was similar to that of nano-LSW-low (**Figures 1D–F**).

### Component Analysis of Nano-LSW

In previous studies, we have confirmed that the main components in LSW-ET include telocinobufagin, bufotaline, cinobufotalin, bufalin, cinobufagin, and resibufogenin, most of them have anticancer activity (Chen et al., 2018). We separated the components of LSW-ET and Nano-LSW using HPLC, and determined the content of their main components. The mass fractions of the main components in LSW-ET and it`s nanoparticles (Nano-LSW-high and Nano-LSW-low) from LSW are denoted as follows (**Supplementary Figure 1** and **Supplementary Table 1**): Ψ-Bufarenogin (0.07%, 0.05%, and 0.01%, respectively), Gamabufotali (0.10%, 0.06%, and 0.01%, respectively), Bufarenogin (0.09%, 0.07%, and 0.02%, respectively), Arenobufagin (0.18%, 0.11%, and 0.02%, respectively), Telocinobufagin (0.15%, 0.10%, and 0.20%, respectively), Bufotaline (0.19%, 0.13%, and 0.02%, respectively), Cinobufotalin (0.18%, 0.17%, and 0.03%, respectively), Bufalin (0.30%, 0.21%, and 0.04%, respectively), Cinobufagin (0.48%, 0.33%, and 0.04%, respectively), Resibufogenin (0.51%, 0.34%, and 0.05%, respectively).

### Nano-LSW-Low Was Much Less Toxicity to Normal Cells *In Vivo*

**Figure 2:**
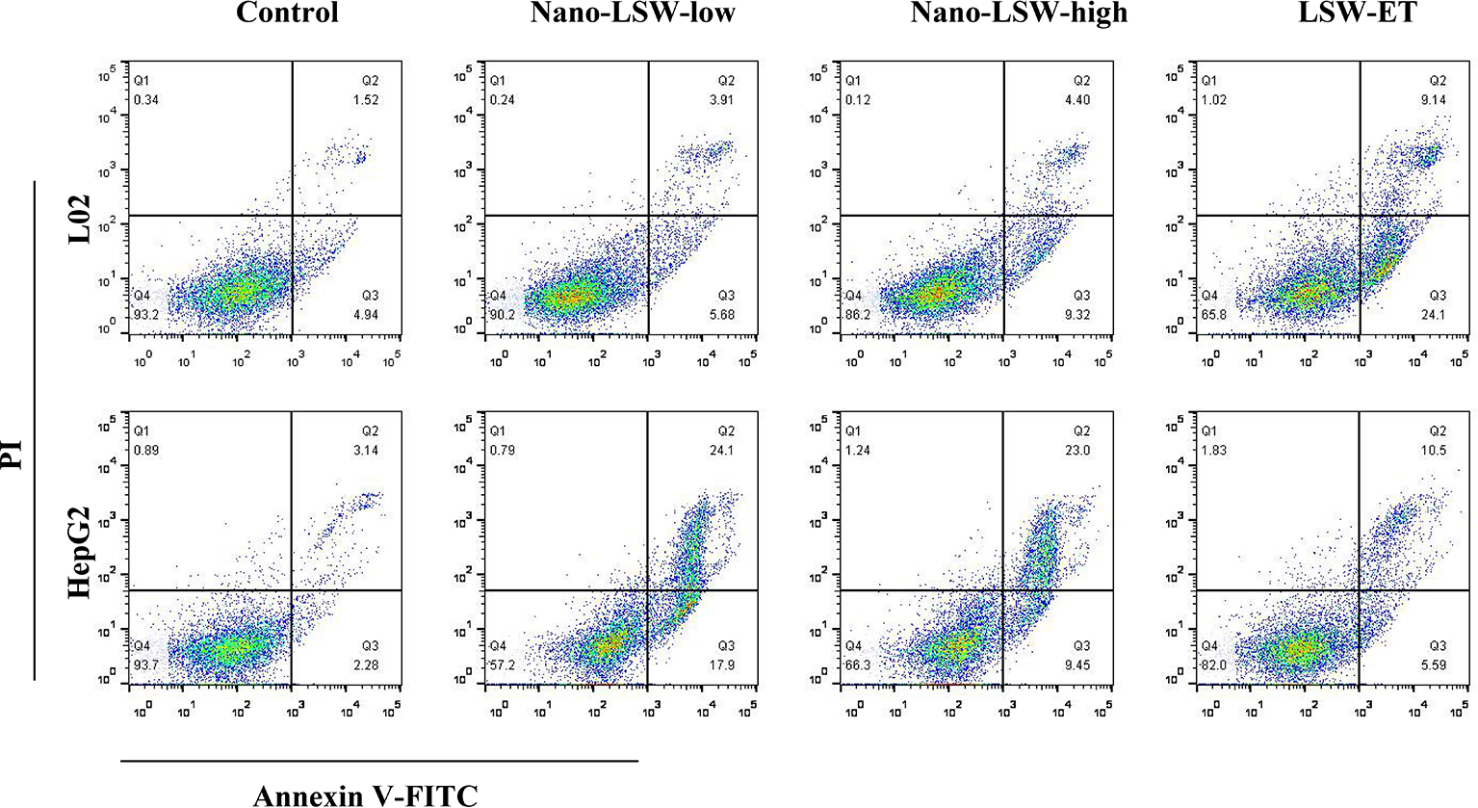
Nano-LSW induced apoptosis in L02 cells and HepG2 cells *in vitro*. Apoptotic cells (L02 cells in top panel; HepG2 cells in bottom panel) were stained with Annexin V-FITC and PI and observed using a fluorescence microscope (magnification 200×) after treatment with or without nano-LSW-low, nano-LSW-high or LSW-ET. Flow cytometry was also used for separating phases of apoptosis after treatment of the drugs mentioned above. After collecting the cells, 400 ul 1x Annexin V binding solution was added to each EP tube. Then, 5 ul Annexin V-FITC staining fluid was added. The solution was gently mixed and then incubated for 15 min at 4°C in the dark. Then, 10 ul PI dye was added. The solution was gently mixed and incubated for 5 min at 4°C in the dark. Then, the sample was immediately examined using a flow cytometer. Top left (Q1): necrotic cells; top right (Q2): late apoptotic cells; bottom left (Q3): live cells; bottom right (Q4): early apoptotic cells.

We conducted an *in vitro* experiment to investigate the effects of nano-LSW-low apoptosis in different cell. As shown in **Figure 2**, the percent of apoptotic L02 cells induced by nano-LSW-low (9.59% early and late apoptosis) and nano-LSW-high (13.72% early and late apoptosis) was significantly decreased compared with that induced by LSW-ET (33.24% early and late apoptosis). Moreover, we found that the number of apoptotic of HepG2 cells was increased by nano-LSW-low (42% early and late apoptosis) and nano-LSW-high (32.45% early and late apoptosis), compared to that of HepG2 cells in the LSW-ET group (16.09% early and late apoptosis).

### Nano-LSW-Low Was Much Less Toxicity to Normal Organs In Vivo

According to our previous investigation (Li et al., 2014), LSW-ET was toxic, so in this study we investigated the acute toxicity of its nanosized materials in animals. As shown in **Supplementary Figure 2**, we investigated the effects of LSW-, LSW-ET, and nano-LSW-low on liver damage in mice using a 14-day acute toxicity test, respectively. Compared with the LSW and LSW-ET groups, the level of inflammatory infiltration in the nano-LSW-low group was minimal. In addition, almost no inflammatory infiltration occurred in the kidney and small intestine of mice exposed to nano-LSW-low alone.

### Oral Uptake of Nano-LSW-Low Phenotypically Relieved Liver Damage in Mice

**Figure 3:**
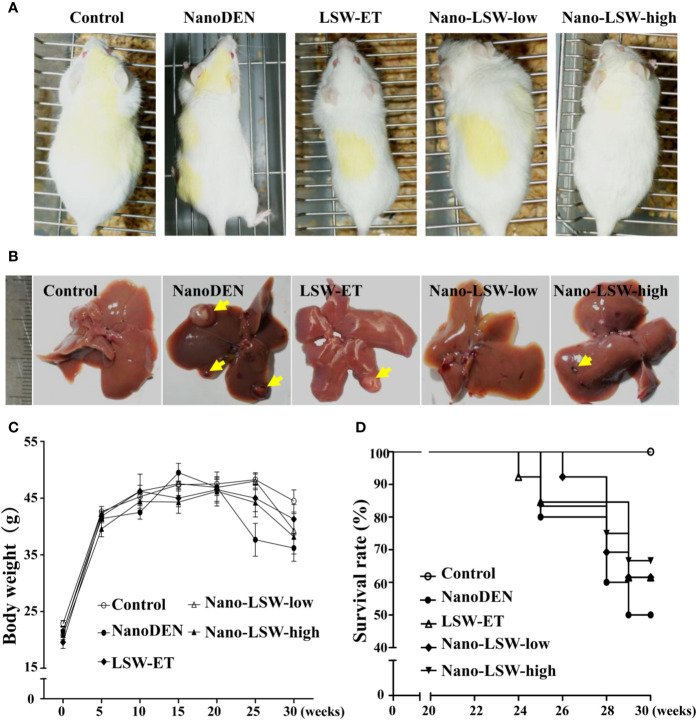
Overview of mice with different treatments. **(A)** General view of the mice. **(B)** General view of the liver samples. The yellow triangle points to a tumor nodule. **(C)** Gain in body weight in different groups during the experiment. **(D)** Survival rates of mice in various groups during liver carcinogenesis.

To explore the antitumor effect of nano-LSW *in vivo*, a nanoDEN-induced mouse model of liver cancer was employed. Liver specimens of the mice in the different groups were harvested at the 10^th^, 15^th^, 20^th^, and 30^th^ week, respectively. As shown in **Figure 3**, there was no difference in the phenotype and diet of the mice within the first 15 weeks of the experiment. However, after the 20^th^ week, mice exposed to nanoDEN alone were thinner and lighter with dull and yellowish hair, compared with the control group and the other treatment groups. Additionally, several tumor nodules appeared on the liver surface in the mice exposed to nanoDEN alone (**Figure 3B**) at the 30^th^ week. In contrast, only a few tumor nodules were present in the livers of nanoDEN-exposed mice after treatment with LSW-ET or nano-LSW-high. Interestingly, no tumor nodules were found in the liver of nanoDEN-exposed mice after treatment with nano-LSW-low. Importantly, the nanoDEN-exposed mice died at about 25 weeks. At 30 weeks, the survival rate of nanoDEN-exposed mice was 50%, whereas the survival rate of mice in LSW-ET group, nano-LSW-low group, and nano-LSW-high group were all above 62%. The results show after drug treatment improved the survival rate of mice exposed to nanoDEN (**Figure 3D**).

**Figure 4:**
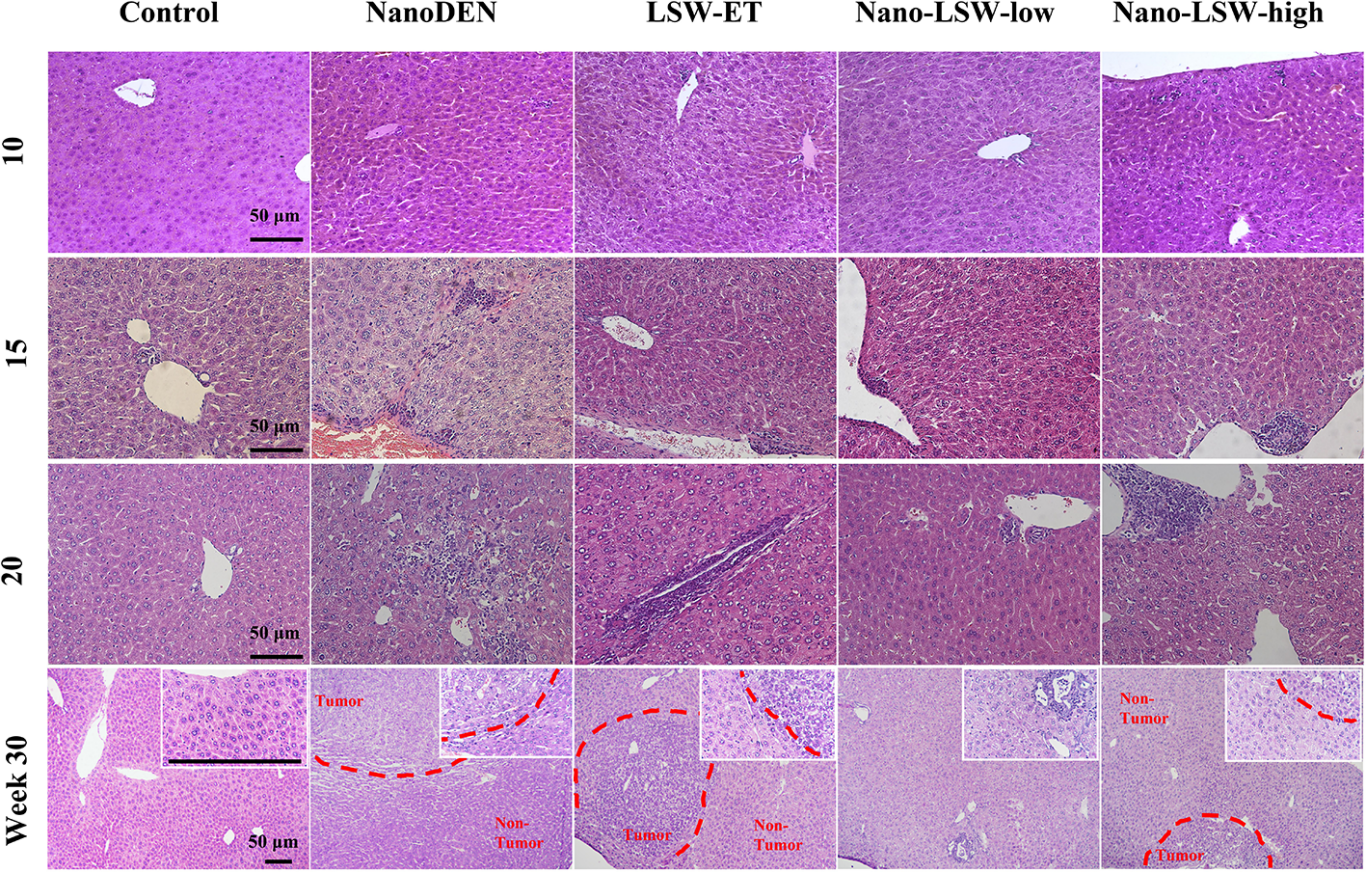
Anti-cancer efficacy of nano-LSW in livers of nanoDEN-treated mice. H&E staining of liver sections in control, nanoDEN, LSW-ET, nano-LSW-low and nano-LSW-high groups at 10, 15, 20, and 30 weeks. Scale bar, 50 µm.

In our previous study, nanoDEN caused severe inflammatory infiltration during liver carcinogenesis. In the present study, we found that the liver tissues of mice exposed to nanoDEN from the 15^th^ week to 30^th^ week exhibited increased severe inflammatory infiltration accompanied by histologic liver disorders (including adipogenesis, fibrosis, tumor; **Figure 4**), compared with the control group. Upon oral administration of LSW-ET, nano-LSW-low, or nano-LSW-high, the histopathological changes in liver mentioned above improved over time. Nano-LSW-low exhibited the best therapeutic effect compared to the other two groups.

### Oral Uptake of Nano-LSW-Low Significantly Attenuated COX-2 Expression in the Livers of nanoDEN-Exposed Mice

Previously, we demonstrated that the expression of multiple critical factors associated with the tumor microenvironment was substantially increased in the livers of nanoDEN-exposed mice (Zhang et al., 2017b). To further evaluate the therapeutic targets of nano-LSW-low, we monitored the changes in these critical factors (including COX-2, TNF-α, β-catenin, HMGB1, PCNA, PPAR-γ, AP-2, Smad-2, and TGF-β1) in the livers of the mice in each group at the 10^th^, 15^th^, 20^th^, and 30^th^ weeks.

**Figure 5:**
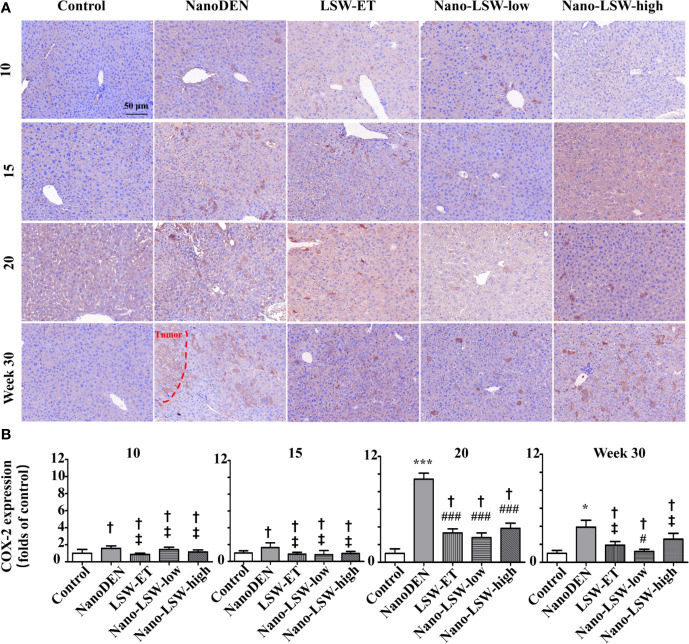
Nano-LSW-low decreased COX-2 expression in livers of nanoDEN-treated mice. **(A)** Representative and **(B)** summary of histochemistry analysis of COX-2 expression. Compared with the control group, ∗, P<0.05; ∗∗∗, P<0.001; †, P > 0.01. Compared with the nanoDEN group, #, P<0.05; ###, P<0.001; ‡, P > 0.01. Scale bar, 50 µm.

Firstly, we investigated the therapeutic effect of nano-LSW-low in the inflammatory microenvironment in the livers of the nanoDEN-exposed mice. As show in **Figure 5**, COX-2 protein expression in the livers of mice exposed to nanoDEN alone (9.42 ± 0.68 and 3.91 ± 0.77 folds of control at the 20^th^ and 30^th^ week, respectively) was greatly increased compared with that in the control group. Compared with the nanoDEN group, oral administration of nano-LSW-low significantly attenuated COX-2 expression (2.78 ± 0.54 and 1.21 ± 0.25 folds of control at 20^th^ and 30^th^ weeks, respectively). However, there was no significant difference in COX-2 expression among the nanoDEN (3.91 ± 0.77 folds of control), LSW-ET (1.90 ± 0.41 folds of control) and nano-LSW-high (2.57 ± 0.66 folds of control) groups at the 30^th^ week.

### Oral Uptake of Nano-LSW-Low Significantly Attenuated PCNA Expression in the Livers of nanoDEN-Exposed Mice

**Figure 6:**
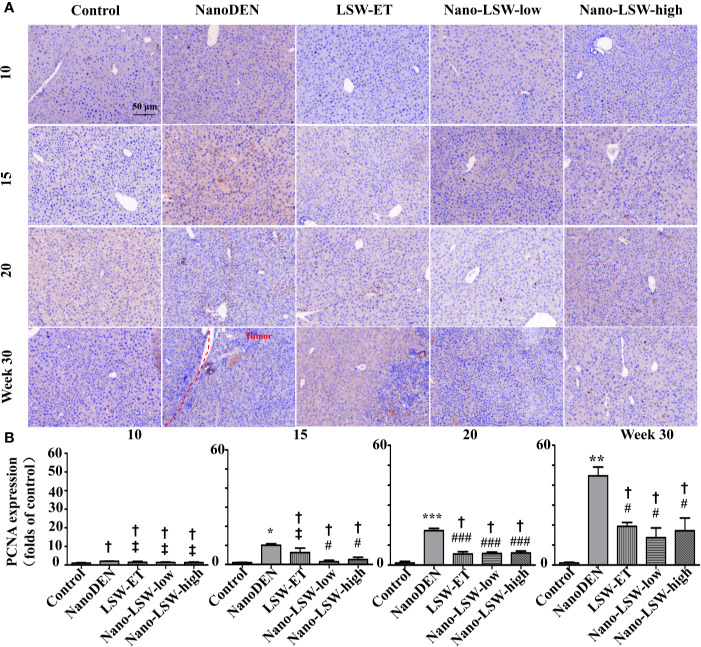
Nano-LSW-low decreased PCNA expression in livers of nanoDEN-treated mice. **(A)** Representative and **(B)** summary of histochemistry analysis of PCNA expression. Compared with the control group, *, P<0.05; ***, P<0.001; ^†^, P > 0.01. Compared with the nanoDEN group, #, P<0.05; ###, P<0.001; ‡, P > 0.01. Scale bar, 50 µm.

Then, we investigated the therapeutic effect of nano-LSW-low in the tumor microenvironment in the livers of nanoDEN-exposed mice. As shown in **Figure 6**, the level of PCNA protein expression in the livers of the mice exposed to nanoDEN alone (17.29 ± 1.09 and 44.66 ± 4.41 folds of control at the 20^th^ and 30^th^ weeks, respectively) was greatly increased compared with that in the control group. Compared to the nanoDEN group, the oral administration of nano-LSW-low significantly attenuated PCNA expression (5.81 ± 0.73 and 13.68 ± 4.91 folds of control at the 20^th^ and 30^th^ week, respectively). In addition, the oral administration of both LSW-ET (5.60 ± 1.13 and 19.36 ± 1.94 folds of control at the 20^th^ and 30^th^ weeks, respectively) and nano-LSW-high (6.07 ± 0.98 and 17.15 ± 6.35 folds of control at the 20^th^ and 30^th^ weeks, respectively) showed similar inhibitory effects on PCNA expression induced by nanoDEN.

### Oral Uptake of Nano-LSW-Low Attenuated Significantly β-catenin Expression in the Livers of nanoDEN-Exposed Mice

Next, we investigated the therapeutic effect of nano-LSW-low on the changes in the nuclear and cytoplasmic localization of β-catenin, which is a key factor of Wnt signaling that regulates the inflammatory response and cell proliferation (Li et al., 2018).

**Figure 7:**
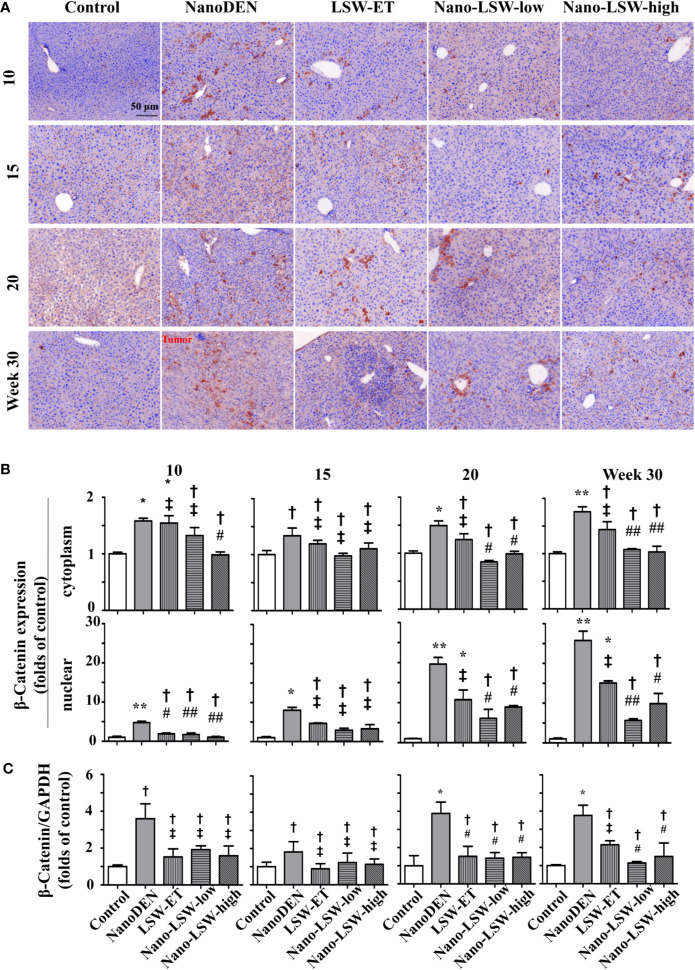
Nano-LSW-low decreased β-catenin expression in livers of nanoDEN-treated mice. **(A)** Representative and **(B)** summary of histochemistry analysis of β-catenin expression. **(C)** β-Catenin mRNA expression in different liver lesions of mice in each group assayed by RT-PCR. Compared with the control group, ∗, P<0.05; ∗∗, P<0.01; †, P > 0.01. Compared with the nanoDEN group, #, P<0.05; ##, P<0.01; ‡, P > 0.01. Scale bar, 50 µm.

As show in **Figure 7**, the accumulation of β-catenin in both the cytoplasm (1.58 ± 0.05, 1.34 ± 0.14, 1.49 ± 0.08, and 1.76 ± 0.09 folds of control, respectively) and nucleus (4.77 ± 0.32, 8.05 ± 0.72, 19.63 ± 1.67 and 25.79 ± 2.32 folds of control, respectively) of hepatocytes gradually increased in the livers of nanoDEN-exposed mice from the 10^th^ week to 30^th^ weeks. Consistent with our previous results, the expression of β-catenin in nanoDEN-exposed mice was higher in normal liver tissue than in tumor tissue (Zhang et al., 2017b). The accumulation of β-catenin in the tumor area was mainly located in the hepatocyte nucleus (19.63 ± 1.67 and 25.79 ± 2.32 folds of control at the 20^th^ and 30^th^ weeks, respectively). As expected, the oral administration of both nano-LSW-low (8.10 ± 0.75 and 5.64 ± 0.41 folds of control at the 20^th^ and 30^th^ weeks, respectively) and nano-LSW-high (8.90 ± 0.34 and 9.89 ± 2.54 folds of control at the 20^th^ and 30^th^ weeks, respectively) significantly attenuated β-catenin nuclear localization in the hepatocytes of nanoDEN-exposed mice (**Figure 7B**). However, the effect of LSW-ET on β-catenin expression in both the cytoplasm (1.24 ± 0.10 and 1.44 ± 0.14 folds of control at the 20^th^ and 30^th^ week, respectively) and nucleus (10.75 ± 2.40 and 15.06 ± 0.54 folds of control at the 20^th^ and 30^th^ week, respectively) of hepatocytes was not as high compared with that of its nanosized materials.

Meanwhile, we detected the effect of the oral uptake of nano-LSW-low on the dynamic changes in *β-catenin* mRNA expression by real-time quantitative PCR. As shown in **Figure 7C**, both LSW-ET (1.52 ± 0.56 folds of control) and its nanosized materials (1.42 ± 0.31 and 1.47 ± 0.26 folds of control) significantly attenuated *β-catenin* mRNA expression in the livers of nanoDEN-exposed (3.88 ± 0.62 folds of control) mice at the 20^th^ week. Furthermore, the level of *β-catenin* mRNA expression restored by nano-LSW-low (1.14 ± 0.08 folds of control) was lower than that restored by LSW-ET (2.17 ± 0.22 folds of control) at the 30^th^ week.

### Oral Uptake of Nano-LSW-Low Significantly Attenuated HMGB1 Expression in the Livers of nanoDEN-Exposed Mice

As another important factor in the pathogenesis of liver cancer, HMGB-1 can inhibit cell apoptosis and promote tumor invasion and metastasis (Kostova et al., 2010). Hence, we also investigated the therapeutic effect of nano-LSW-low on the changes in the nuclear and cytoplasmic localization of HMGB1 during tumorigenesis.

**Figure 8:**
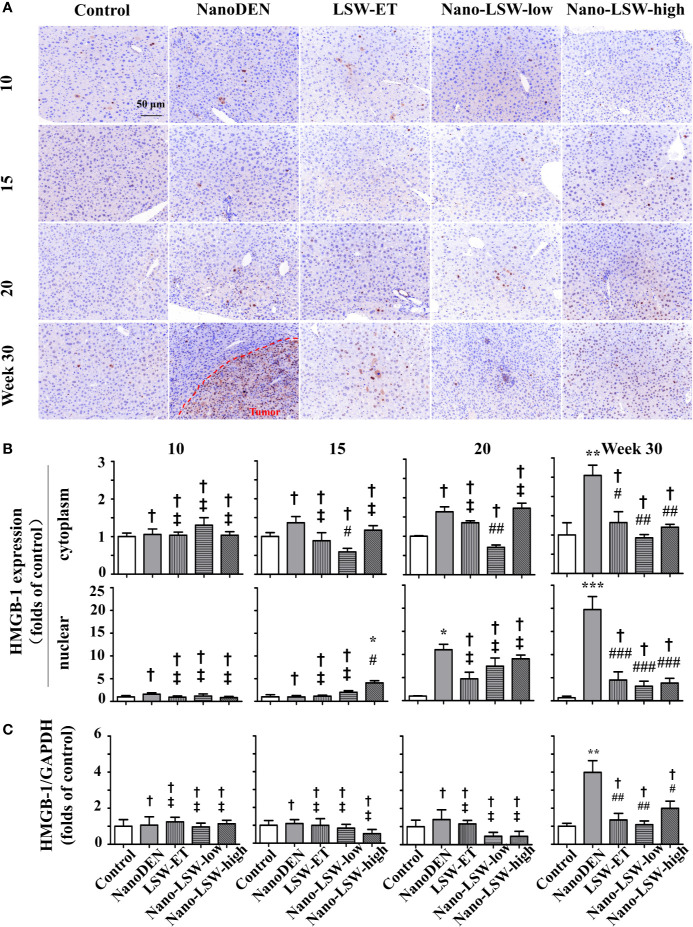
Nano-LSW-low decreased HMGB-1 expression in livers of nanoDEN-treated mice. **(A)** Representative and **(B)** summary of histochemistry analysis of HMGB-1 expression. **(C)** HMGB-1 mRNA expression in different liver lesions of mice in each group assayed by RT-PCR. Compared with the control group, ∗, P<0.05; ∗∗, P<0.01; ∗∗∗, P<0.001; †, P > 0.01. Compared with the nanoDEN group, #, P<0.05; ##, P<0.01; ###, P<0.001; ‡, P > 0.01. Scale bar, 50 µm.

As shown in **Figures 8A, B**, the accumulation of HMGB1 in both the cytoplasm (1.05 ± 0.15, 1.36 ± 0.16, 1.63 ± 0.13 and 2.55 ± 0.27 folds of control, respectively) and nucleus (1.58 ± 0.31, 0.97 ± 0.30, 11.02 ± 1.17 and 19.73 ± 2.80 folds of control, respectively) of hepatocytes gradually increased in the livers of nanoDEN-exposed mice from the 10^th^ to 30^th^ weeks. HMGB1 mainly accumulated in the hepatocytes in the tumor area (**Figure 8A**).

As expected, the oral administration of nano-LSW-low (0.59 ± 0.09, 0.71 ± 0.06 and 0.92 ± 0.09 folds of control at the 15^th^, 20^th^, and 30^th^ weeks, respectively) significantly attenuated the high cytoplasmic HMGB1 localization in the hepatocytes of the nanoDEN-exposed mice (**Figures 8A, B****)**. However, compared to the nanoDEN group, both LSW-ET and nano-LSW-high exhibited a significant inhibitory effect on HMGB1 expression in both the cytoplasm (1.32 ± 0.28 and 1.20 ± 0.07 folds of control) and nucleus (4.49 ± 1.78 and 3.84 ± 1.02 folds of control, respectively) of the hepatocytes exclusively at the 30^th^ week.

### Changes in mRNA Levels of Multiple Critical Factors for the Tumor Microenvironment in the Livers of nanoDEN-Exposed Mice After Oral Uptake of Nano-LSW-Low

To further assess the hepatic microenvironment observed above, we monitored changes in the mRNA of multiple critical factors associated with inflammation (*COX-2* and *TNF-α*), pimelosis (*PPAR-γ* and *AP-2*), and fibrosis (*Smad-2* and *TGF-β1*) during oncogenesis.

First, as shown in **Supplementary Figures 3A, B**, we observed that the mRNA expression of both *COX-2* (115 ± 15%, 222 ± 82%,560 ± 97% and 306 ± 62% of control, respectively) and *TNF-α* (81 ± 17%, 175 ± 32%, 365 ± 24% and 304 ± 56% of control, respectively) gradually increased in the livers of nanoDEN-exposed mice from the 10^th^ to 30^th^ weeks. According to the data shown in **Supplementary Figures 3A, B**, *COX-2* and *TNF-α* mRNA expression reached a peak level in the livers of nanoDEN-exposed mice at the 20^th^ week. As expected, these peak levels were intentionally suppressed by LSW-ET *(COX-2*: 85 ± 29% of control; *TNF-α*: 197 ± 47% of control) and its nanosized materials (*COX-2*: 63 ± 21% and 144 ± 55% of control for nano-LSW-low; *TNF-α*: 98 ± 19% and 123 ± 13% of control for nano-LSW-high), respectively. However, nano-LSW-low most effectively achieved peak levels compared to the other two therapeutic agents.

Then, as shown in **Supplementary Figures 3C, D**, an increasing trend in the mRNA expression of both *PPAR-γ* and *AP-2* was noted in the livers of nanoDEN-exposed mice from the 10^th^ to 30^th^ weeks. Unexpectedly, the oral administration of LSW-ET and its nanosized materials could not significantly attenuate this increasing trend.

Finally, we monitored the changes in the *Smad-2* and *TGF-β1* mRNA levels during oncogenesis. As shown in **Supplementary Figures 3E, F**, the mRNA expression levels of *Smad-2* (84 ± 12%, 94 ± 19%, 247 ± 32%, and 344 ± 24% of control, respectively) and *TGF-β1* (91 ± 10%, 121 ± 7%, 224 ± 21%, and 245 ± 45% of control, respectively) were gradually increased in the livers of nanoDEN-exposed mice from the 10^th^ to 30^th^ weeks. As expected, these increased levels were intentionally suppressed by LSW-ET (*Smad-2*: 165 ± 18% and 172 ± 7% of control; *TGF-β1*: 130 ± 29% and 130 ± 26% of control), nano-LSW-low (*Smad-2*: 85 ± 19% and 86 ± 33% of control; *TGF-β1*: 87 ± 13% and 124 ± 16% of control) and nano-LSW-high (*Smad-2*: 126 ± 30% and 91 ± 33% of control; *TGF-β1*: 156 ± 8% and 166 ± 11% of control) at the 20^th^ and 30^th^ weeks, respectively. Hence, nano-LSW-low exhibited the best effects on the increased levels of fibrosis-associated factors compared to those of the other two therapeutic agents.

## Discussion

There are few drugs that can be used clinically to treat liver cancer, and the long-term use of chemotherapy drugs is associated with strong toxic side effects in patients. Previous studies have shown that DEN can induce liver damage, and lipid nanoparticle-packed DEN can generate liver cancer in mice effectively (Zhang et al., 2017b). Therefore, the study of high-efficiency liver cancer treatment drugs that exhibit low (or no) toxicity is crucial (Dhanasekaran et al., 2010). It has been reported that the alcohol extracted portion of LSW-ET is the main effective portion of LSW against tumors (Chen et al., 2018). However, LSW contains realgar and venenum bufonis, whichh has been queried for its safety (Zhang et al., 2017a). In order to reduce the damage of toxic substances to the body, it is very important to develop a drug targeting method. It is well known that nano-delivery system can improve drug targeting and increase solubility, as lipid nanoparticle can enter human blood through oral, transdermal and other routes and distribute to specific organs and cells (Harisa et al., 2018). Therefore, we placed the LSW-ET product into the nano drug-loading system to produce a nano-LSW preparation with a particle size of approximately 200 nm, a negative potential, and stable release *in vitro* (**Figure 1**).

The experiment was carried out by an *in vitro* cell experiment. Our results showed that nano-LSW induced significantly more apoptosis in HepG2 cells compared with LSW-ET. We hypothesize that nanoparticles with small size and a lipid structure are beneficial to be taken up by cells. With the higher intake of nano-LSW, the expression of PCNA, HMGB1 and other related tumor proliferation and migration factors in cells may be altered. In addition, the microenvironment conditions of HepG2 cells were also likely altered. Furthermore, the tumor cells cannot divide and proliferate, leading to apoptosis (Qin et al., 2010; Shi et al., 2014). Certainly, negative surface charge for lipid nanoparticles can cause lower cytotoxicity than cationic surface charge for nanoparticles (Fröhlich, 2012). Meanwhile, the less toxic components of nano-LSW-low are less toxic to L02 cells.

Next, we further explored the anti-tumor efficacy of nano-LSW-low in mice. We found that nano-LSW-low is less toxic to normal tissues than LSW-ET in an acute toxicity study (**Supplementary Figure 2**). Simultaneously, nano-LSW has a good therapeutic effect on liver cancer induced by nanoDEN. The reason is that nanoliposomes exhibit good passive targeting and can selectively concentrate in the rich organizers of the reticuloendothelial system in the body (Sasikumar and Kamalasanan, 2017). Moreover, because of the enhanced permeability and retention effect (EPR), nanoliposomes build accumulation in tumors preferentially. In addition, there is no structurally functional lymphatic system in the tumor tissue; thus, the nanoparticles are rarely cleared, and the drug is retained in the tumor tissue (Dong and Mumper, 2010). In previous studies, we demonstrated that lipid nanoparticles can enhance the liver targeting of drug. Therefore, we hypothesize that nano-LSW also exhibits good liver targeting.

Furthermore, our results demonstrated that the high expression of inflammatory (COX-2 and TNF-α), steatosis (PPAR-γ and AP-2), fibrosis (Smad-2 and TGF-β1), PCNA and HMGB1 proteins in tumors was downregulated after treatment with nano-LSW (**Supplementary Figure 3**; **Figures 6** and **8**). In liver cancer, high expression of the inflammatory factor COX-2 activates the nuclear expression of β-catenin. Thus, ECM accumulates in hepatic stellate cells, and hepatic stellate cells transform into fibroblasts, thereby activating oncogenic pathways, such as the TGF-β/Smad pathway (Zulehner et al., 2010; Cai et al., 2015). Moreover, the upregulation of HMGB1 and PCNA promotes the proliferation and migration of cancer cells. We proposed a hypothesis the high expression of related proteins in the process of tumor development is downregulated after the administration of nano-LSW, which leads to changes in the microenvironment of tumor cells. However, tumor cells cannot adapt to these changes in the environment and eventually die, but normal liver cells are not sensitive to these changes in the environment. There is another possibility, because we administer the liver cancer before it forms, nano-LSW inhibits the formation of tumor microenvironment and delays the development of liver cancer by inhibiting the high expression of related proteins.

Interestingly, we found that nano-LSW-low has better anticancer effects than nano-LSW-high. We hypothesized that the concentration of nano-LSW-high exceeded the maximum effective concentration of the drug. More investigations could be performed to explore the dose-effect relationship of drugs.

## Conclusion

Overall, we demonstrated that nano-LSW-low exhibits low toxicity to normal organs in mice. Meanwhile, nano-LSW-low exhibited excellent efficacy in preventing liver cancer by interfering with the tumor microenvironment during oncogenesis. Nano-LSW-low might be a promising drug to prevent and treat liver cancer.

## Data Availability Statement

The raw data supporting the conclusions of this article will be made available by the authors, without undue reservation, to any qualified researcher.

## Ethics Statement

The animal study was reviewed and approved by the Committee on the Ethics of Animal Experiments of the South-Central University for Nationalities, Wuhan, China (2012-SCUEC-AEC-002).

## Author Contributions

JH, X-ZC, Y-SL, and H-BT conceived and designed the experiments. JH, X-ZC, and Y-SL performed the experiments. X-ZC, Y-GP, WZ, Y-SL, and H-BT analyzed the data. JH, X-ZC, H-BT, and Y-SL contributed reagents/materials/analysis tools. JH, Y-GP, X-ZC, WZ, H-BT, and Y-SL wrote the paper.

## Funding

This work was supported by the National Natural Science Foundation of China (81573887, 81673711).

## Conflict of Interest

The authors declare that the research was conducted in the absence of any commercial or financial relationships that could be construed as a potential conflict of interest.

## References

[B1] Aires A. Ocampo S. M. Simões B. M. Josefa Rodríguez M. Cadenas J. F. Couleaud P. (2016). Multifunctionalized iron oxide nanoparticles for selective drug delivery to CD44-positive cancer cells. Nanotechnology 27 (6), 065103. 10.1088/0957-4484/27/6/065103 26754042

[B2] Bhattacharyya S. Kudgus R. A. Bhattacharya R. Mukherjee P. (2011). Inorganic nanoparticles in cancer therapy. Pharm. Res. 28 (2), 237–259. 10.1007/s11095-010-0318-0 21104301 PMC3072278

[B3] Cai X. Li F. Zhang Q. Xu M. Qu Y. Wan X. (2015). Peritumoral ductular reaction is related to nuclear translocation of β-catenin in hepatocellular carcinoma. BioMed. Pharmacother. 76, 11–16. 10.1016/j.biopha.2015.10.017 26653544

[B4] Chen X. Z. Zhang W. K. Tang H. B. (2018). The Ethanol Supernatant Extracts of Liushenwan Could Alleviate Nanodiethylnitrosamine-Induced Liver Cancer in Mice. Can. J. Gastroenterol. 2018, 6934809. 10.1155/2018/6934809 PMC6178154 30356380

[B5] Dhanasekaran R. Kooby D. A. Staley C. A. Kauh J. S. Khanna V. Kim H. S. (2010). Comparison of conventional transarterial chemoembolization (TACE) and chemoembolization with doxorubicin drug eluting beads (DEB) for unresectable hepatocelluar carcinoma (HCC). J. Surg. Oncol. 101 (6), 476–480. 10.1002/jso.21522 20213741

[B6] Dong X. Mumper R. J. (2010). Nanomedicinal strategies to treat multidrug-resistant tumors: current progress. Nanomed. (Lond) 5 (4), 597–615. 10.2217/nnm.10.35 PMC2925023 20528455

[B7] Fröhlich E. (2012). The role of surface charge in cellular uptake and cytotoxicity of medical nanoparticles. Int. J. Nanomed. 7, 5577–5591. 10.2147/ijn.s36111 PMC3493258 23144561

[B8] Harisa G. I. Badran M. M. Alanazi F. K. Attia S. M. (2018). An overview of nanosomes delivery mechanisms: trafficking, orders, barriers and cellular effects. Artif. Cells Nanomed. Biotechnol. 46 (4), 669–679. 10.1080/21691401.2017.1354301 28701048

[B9] Havrdova M. Polakova K. Skopalik J. Vujtek M. Mokdad A. Homolkova M. (2014). Field emission scanning electron microscopy (FE-SEM) as an approach for nanoparticle detection inside cells. Micron (Oxford Engl. 1993) 67, 149–154. 10.1016/j.micron.2014.08.001 25173605

[B10] Jiang J. W. Chen X. H. Ren Z. Zheng S. S. (2019). Gut microbial dysbiosis associates hepatocellular carcinoma via the gut-liver axis. Hepatob. Pancreat Dis. Int. 18 (1), 19–27. 10.1016/j.hbpd.2018.11.002 30527903

[B11] Khalid O. Bouneva I. (2011). Management of hepatocellular carcinoma: treatment options and indications for orthotopic liver transplantation. Mo Med. 108 (4), 264–268. 21905443 PMC6188415

[B12] Kostova N. Zlateva S. Ugrinova I. Pasheva E. (2010). The expression of HMGB1 protein and its receptor RAGE in human malignant tumors. Mol. Cell Biochem. 337 (1-2), 251–258. 10.1007/s11010-009-0305-0 19876719

[B13] Li Y. S. Wang J. X. Jia M. M. Liu M. Li X. J. Tang H. B. (2012). Dragon’s Blood Inhibits Chronic Inflammatory and Neuropathic Pain Responses by Blocking the Synthesis and Release of Substance P in Rats. J. Pharmacol. Sci. 118 (1), 43–54. 10.1254/jphs.11160FP 22198006

[B14] Li X. J. Jia M. M. Li Y. S. Yang Y. L. Mao X. Q. Tang H. B. (2014). Involvement of substance p/neurokinin-1 receptor in the analgesic and anticancer activities of minimally toxic fraction from the traditional Chinese medicine Liu-Shen-Wan in vitro. Biol. Pharm. Bull. 37 (3), 431–438. 10.1248/bpb.b13-00794 24366059

[B15] Li Y. S. Leng C. L. Chen M. T. Zhang W. K. Li X. J. Tang H. B. (2016a). Mouse hepatic neoplasm formation induced by trace level and low frequency exposure to diethylnitrosamine through beta-catenin signaling pathway. Toxicol. Res. (Camb) 5 (1), 210–223. 10.1039/c5tx00317b 30090338 PMC6062358

[B16] Li Y. S. Leng C. L. Chen M. T. Zhang W. K. Li X. J. Tang H. B. (2016b). Mouse hepatic neoplasm formation induced by trace level and low frequency exposure to diethylnitrosamine through β-catenin signaling pathway. Toxicol. Res. (Camb) 5 (1), 210–223. 10.1039/c5tx00317b 30090338 PMC6062358

[B17] Li X. J. Huang F. Z. Wan Y. Li Y. S. Zhang W. K. Xi Y. (2018). Lipopolysaccharide Stimulated the Migration of NIH3T3 Cells Through a Positive Feedback Between beta-Catenin and COX-2. Front. Pharmacol. 9, 1487. 10.3389/fphar.2018.01487 30618773 PMC6305731

[B18] Liang W. L. Xiao L. Gu H. W. Li X. J. Li Y. S. Zhang W. K. (2019). Solid lipid nanoparticle induced apoptosis of macrophages via a mitochondrial-dependent pathway in vitro and in vivo. Int. J. Nanomed. 14, 3283–3295. 10.2147/ijn.s200395 PMC6511261 31123400

[B19] Lin J. Wu J. F. Zhang Q. Zhang H. W. Cao G. W. (2014). Virus-related liver cirrhosis: molecular basis and therapeutic options. World J. Gastroenterol. 20 (21), 6457–6469. 10.3748/wjg.v20.i21.6457 24914367 PMC4047331

[B20] Ma H. Y. Kou J. P. Wang J. R. Yu B. Y. (2007). Evaluation of the anti-inflammatory and analgesic activities of Liu-Shen-Wan and its individual fractions. J. Ethnopharmacol. 112 (1), 108–114. 10.1016/j.jep.2007.02.008 17368990

[B21] Piktel E. Niemirowicz K. Wątek M. Wollny T. Deptuła P. Bucki R. (2016). Recent insights in nanotechnology-based drugs and formulations designed for effective anti-cancer therapy. J. Nanobiotechnol. 14 (1), 39. 10.1186/s12951-016-0193-x PMC4881065 27229857

[B22] Qin X. G. Hua Z. Shuang W. Wang Y. H. Cui Y. D. (2010). Effects of matrine on HepG2 cell proliferation and expression of tumor relevant proteins in vitro. Pharm. Biol. 48 (3), 275–281. 10.3109/13880200903104101 20645813

[B23] Sasikumar A. Kamalasanan K. (2017). Nanomedicine for prostate cancer using nanoemulsion: A review. J. Control Rel. 260, 111–123. 10.1016/j.jconrel.2017.06.001 28583444

[B24] Shi W. Su L. Li Q. Sun L. Lv J. Li J. (2014). Suppression of toll-like receptor 2 expression inhibits the bioactivity of human hepatocellular carcinoma. Tumour Biol. 35 (10), 9627–9637. 10.1007/s13277-014-2268-3 24964964

[B25] Siegel R. Ma J. Zou Z. Jemal A. (2014). Cancer statistics 2014. CA Cancer J. Clin. 64 (1), 9–29. 10.3322/caac.21208 24399786

[B26] Tai C. S. Lin Y. R. Teng T. H. Lin P. Y. Tu S. J. Chou C. H. (2017). Haptoglobin expression correlates with tumor differentiation and five-year overall survival rate in hepatocellular carcinoma. PLoS One 12 (2), e0171269. 10.1371/journal.pone.0171269 28158312 PMC5291462

[B27] Valery P. C. Laversanne M. Clark P. J. Petrick J. L. McGlynn K. A. Bray F. (2018). Projections of primary liver cancer to 2030 in 30 countries worldwide. Hepatol. 67 (2), 600–611. 10.1002/hep.29498 PMC5832532 28859220

[B28] Zhang M. H. Chen J. Q. Guo H. M. Li R. T. Gao Y. Q. Tian Y. (2017a). Combination of LC/MS and GC/MS based metabolomics to study the hepatotoxic effect of realgar nanoparticles in rats. Chin. J. Nat. Med. 15 (9), 684–694. 10.1016/s1875-5364(17)30098-5 28991530

[B29] Zhang W. K. Gu H. W. Li X. J. Li Y. S. Tang H. B. Tian G. H. (2017b). The dark side of “the force” - lipid nanoparticles enhance the oncogenesis of diethylnitrosamine and result in liver cancer in mice. Nanomedicine 13 (2), 701–711. 10.1016/j.nano.2016.09.017 27729235

[B30] Zhang H. Y. Wang H. L. Zhong G. Y. Zhu J. X. (2018a). Molecular mechanism and research progress on pharmacology of traditional Chinese medicine in liver injury. Pharm. Biol. 56 (1), 594–611. 10.1080/13880209.2018.1517185 31070528 PMC6282438

[B31] Zhang M. T. Ye X. X. Lan W. Yang Y. L. Wu T. Li Y. S. (2018b). Strategic Combination of Isocratic and Gradient Elution for Simultaneous Separation of Polar Compounds in Traditional Chinese Medicines by HPLC. J. Anal. Methods Chem. 2018, 7569283. 10.1155/2018/7569283 29744235 PMC5884029

[B32] Zhao X. Chen Q. Li Y. Tang H. Liu W. Yang X. (2015a). Doxorubicin and curcumin co-delivery by lipid nanoparticles for enhanced treatment of diethylnitrosamine-induced hepatocellular carcinoma in mice. Eur. J. Pharm. Biopharm. 93, 27–36. 10.1016/j.ejpb.2015.03.003 25770771

[B33] Zhao X. Chen Q. Liu W. Li Y. Tang H. Liu X. (2015b). Codelivery of doxorubicin and curcumin with lipid nanoparticles results in improved efficacy of chemotherapy in liver cancer. Int. J. Nanomed. 10, 257–270. 10.2147/ijn.s73322 PMC4284012 25565818

[B34] Zulehner G. Mikula M. Schneller D. van Zijl F. Huber H. Sieghart W. (2010). Nuclear beta-catenin induces an early liver progenitor phenotype in hepatocellular carcinoma and promotes tumor recurrence. Am. J. Pathol. 176 (1), 472–481. 10.2353/ajpath.2010.090300 20008139 PMC2797905

