# Supplementary material for: Nanoparticle-Encapsulated Liushenwan Could Treat Nanodiethylnitrosamine-Induced Liver Cancer in Mice by Interfering With Multiple Critical Factors for the Tumor Microenvironment
Source: Front Pharmacol. 2020 Jul 10;11:1052. doi: 10.3389/fphar.2020.01052 (PMC7365909; doi:10.3389/fphar.2020.01052)
Supplement: Supplementary file 1 [file DataSheet_1.pdf]

Supplementary Materials

Nanoparticle-encapsulated Liushenwan could treat nanodiethylnitrosamine-induced liver cancer in mice by interfering with multiple critical factors for the tumor microenvironment

Jing Hong[1,#], Xi-Zhen Chen[1,#], You-Gong Peng[2,#], Wei Kevin Zhang[1], He-Bin Tang[1,] *, Yu-Sang Li[1,]

[*]**Corresponding author:**

Yu-Sang Li, liys2006@mail.scuec.edu.cn, tel/0086-27-67841196;

He-Bin Tang: hbtang2006@mail.scuec.edu.cn, tel/0086-27-67842332;

1. Immunohistochemical of the liver tissues

In brief, the paraffin-embedded liver tissue sections were deparaffinized and hydrated, followed by antigen was retrieved by citric acid buffer (pH = 6.0) microwave antigen retrieval, and the endogenous peroxidase was blocked with a 3% H_2O_2. Subsequently, the sections were blocked with 5% BSA (Cat. No. 36106ES25; Yeasen), then incubated with primary antibodies against PCNA (1:6000), COX-2 (1:200), β-catenin (1:200), HMGB-1 (1:1000), and secondary antibody, respectively. After color development with 3,3'-diaminobenzidine tetrahydrochloride, the sections were counterstained with hematoxylin and mounted using coverslips with aqueous mounting media. Finally, the spectral optical density of the stained sections was automatically acquired from 420 to 720 nm in 10 nm increments with a CRi Nuance Multispectral Imaging System (Cambridge Research and Instrumentation Inc., Woburn, MA, USA) as described in our previous study (Li et al., 2012).

Spectral unmixing for each image was performed using Nuance software (v3.0.2) and pure spectral libraries of individual chromogens. The DAB single-channel image and hematoxylin single-channel image are obtained by spectral decomposition. The

total signal (optical density) of marker of co-localization in DAB image is the total expression of protein-positive regions. The total signals (optical density) of marker of co-localization in DAB image and hematoxylin image are the protein expression level located in the nucleus. The difference between the DAB channel and hematoxylin channel protein expression level is used as the cytoplasmic expression. The measured background noise was subtracted from each image. Three equal-sized fields were chosen at random to quantify the expression of the corresponding protein in each image.

Li, Y.S., Wang, J.X., Jia, M.M., Liu, M., Li, X.J., and Tang, H.B. (2012). Dragon's Blood Inhibits Chronic Inflammatory and Neuropathic Pain Responses by Blocking the Synthesis and Release of Substance P in Rats. *J Pharmacol Sci* 118(1), 43-54. doi: 10.1254/jphs.11160FP.

2. Supplementary Figures and its legends

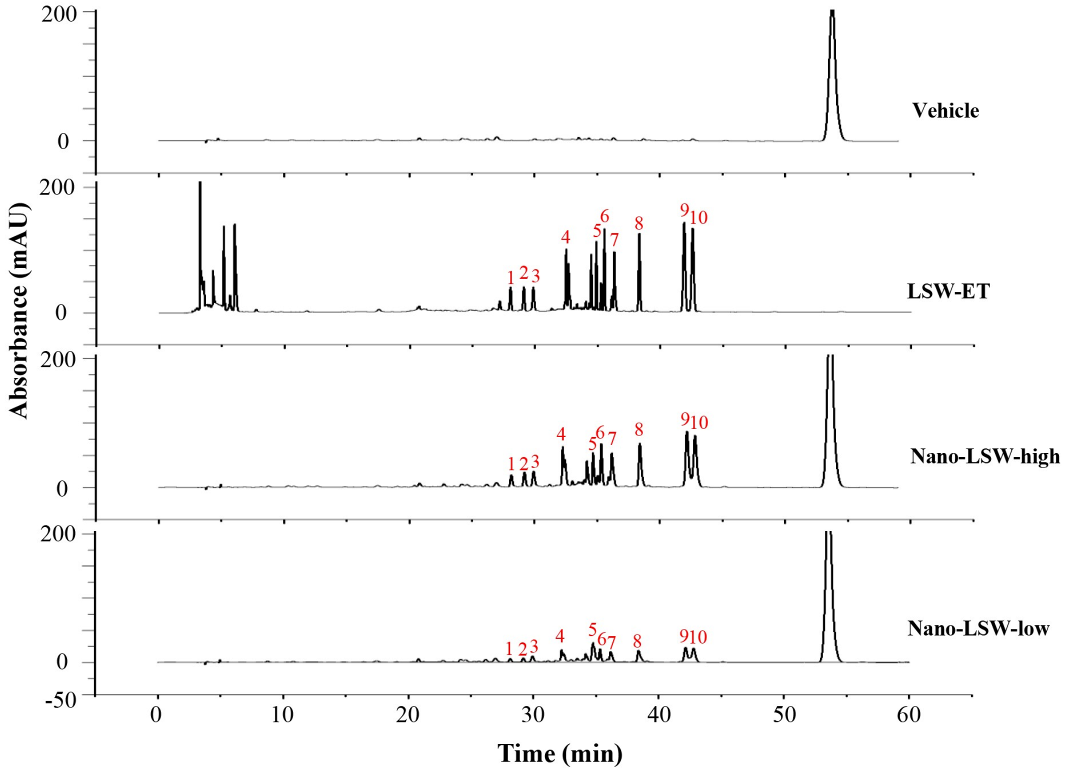

Supplementary Figure 1. Separation of nanosized materials by using HPLC. 1: Ψ-Bufarenogin; 2: Gamabufotali; 3: Bufarenogin; 4: Arenobufagin; 5: Telocinobufagin; 6: Bufotaline; 7: Cinobufotalin; 8: Bufalin; 9: Cinobufagin; 10: Resibufogenin.

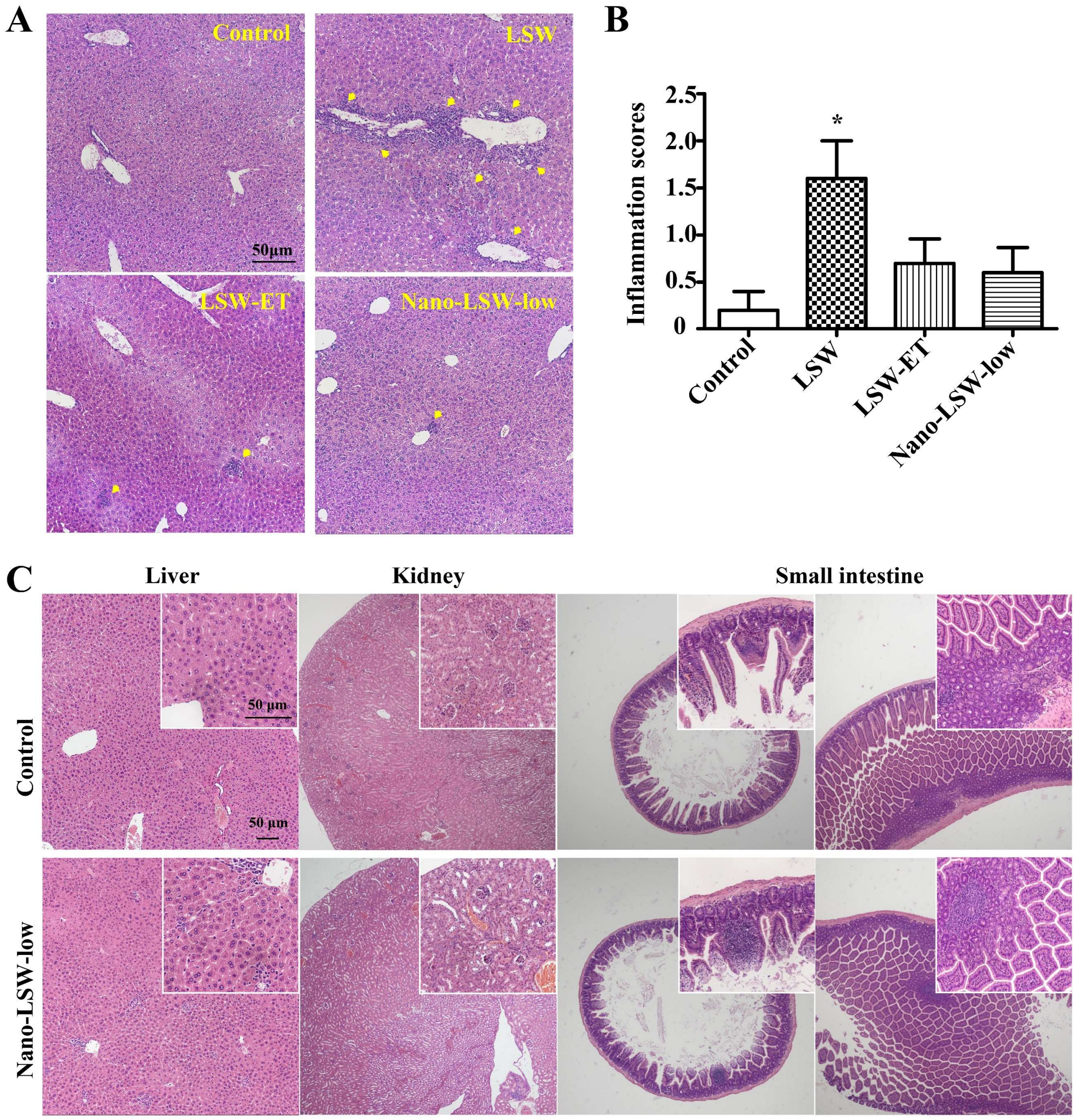

Supplementary Figure 2. Nano-LSW-low has low toxicity to normal organs. (A) Histological analysis of livers from the control group, LSW group, LSW-ET, and nano-LSW-low group at the 14th day. The yellow triangle pointed to inflammation infiltration. (B) Scores of inflammation of livers samples. (C) H&E staining analysis of liver, kidney and small intestine of control group and nano-LSW-low group.

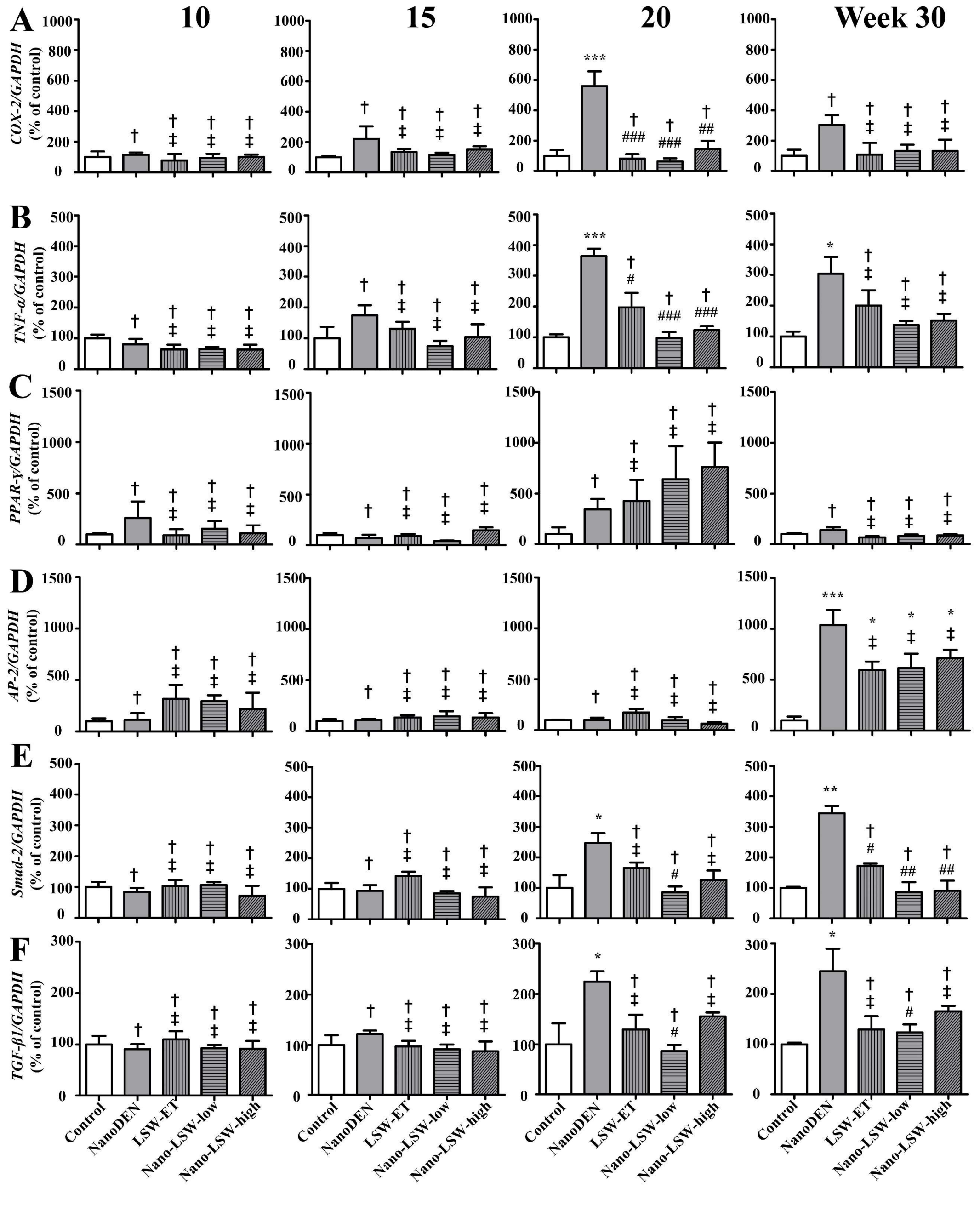

Supplementary Figure 3. Messenger RNA changes of associated with the inflammation, steatosis, fibrosis in mice. (A, B, C, D, E, F) The mRNA expression of COX-2, TNF-α, PPAR-γ, AP-2, Smad-2, TGF-β1, respectively. Compared with the control group, ∗, P<0.05; ∗∗, P<0.01; †, P > 0.01. Compared with the nanoDEN group, #, P<0.05; ##, P<0.01; ‡, P > 0.01. The DAB images of PCNA in the Week 30 group.

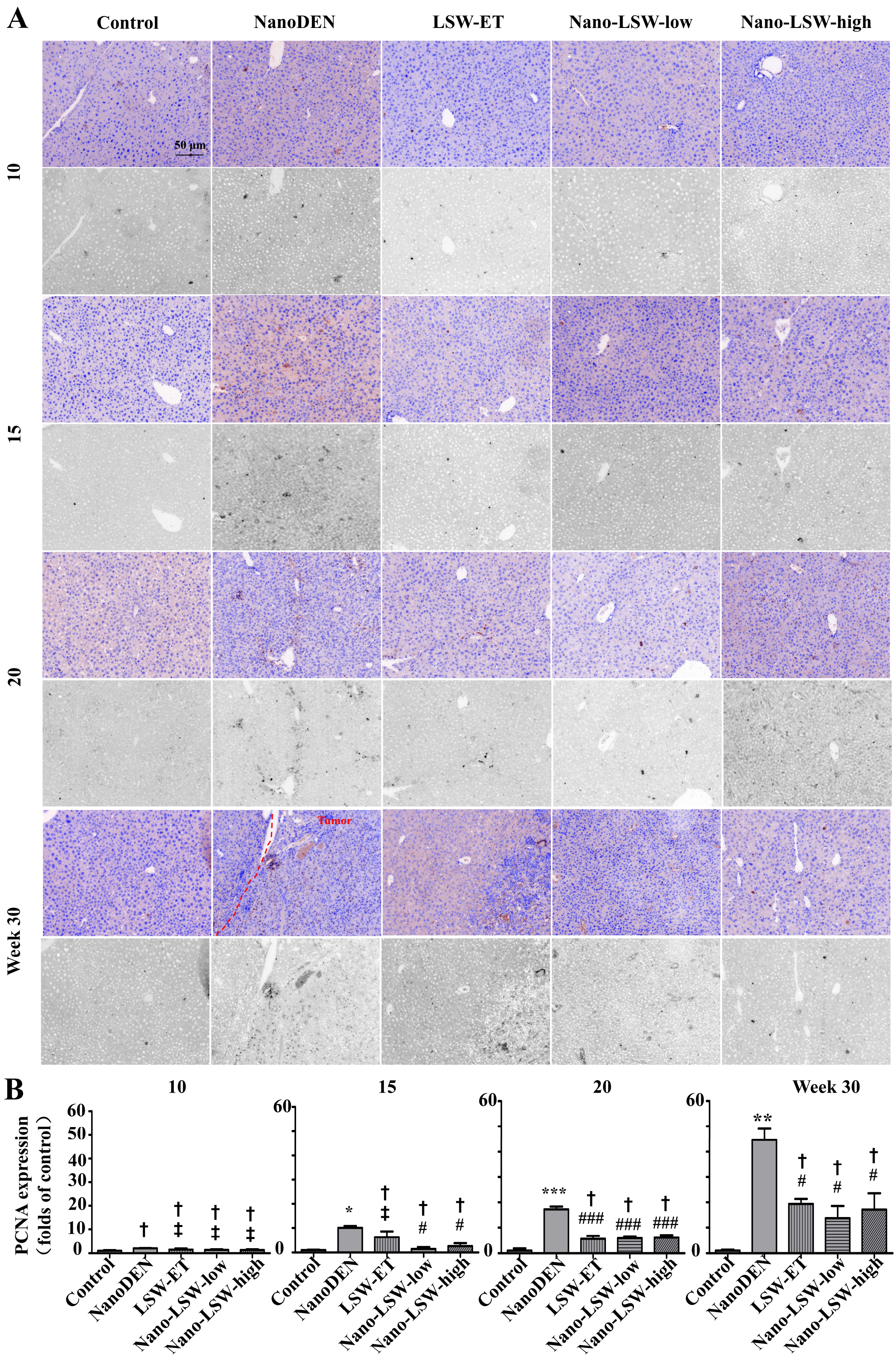

Supplementary Figure 4. The DAB images of PCNA in the Week 10-30 groups.

Supplementary Tables

Table 1. Component analysis of LSW-ET

No	Ret Time (min)	Content (%)	Compound
1	28.20	0.07	Ψ-Bufarenogin
2	29.25	0.10	Gamabufotali
3	29.99	0.09	Bufarenogin
4	32.27	0.18	Arenobufagin
5	34.68	0.15	Telocinobufagin
6	35.35	0.19	Bufotaline
7	36.19	0.18	Cinobufotalin
8	38.44	0.30	Bufalin
9	42.23	0.48	Cinobufagin
10	42.87	0.51	Resibufogenin

Table 2. Summary of the primers designed and used in this study

Gene	Forward primer (5' to 3')	Reverse primer (5' to 3')
**β-catenin**	CCTAGCTGGTGGACTGCAGAA	CACCACTGGCCAGAATGATGA
**PPAR-γ**	CGCTGATGCACTGCCTATGA	AGAGGTCCACAGAGCTGATTCC
**TEG-β1**	GTGTGGAGCAACATGTGGAACTCTA	CGCTGAATCGAAGCCCTGTA
**Smad-2**	AACCCGAATGTGCACATAAGAA	ATGCTTGAGCATCGCACTGAA
**COX-2**	CTGGAACATGGACTCACTCAGTTTG	AGGCCTTTGCCACTGCTTGTA
**TNF-α**	ACCCTCACACTCAGATCATCTTC	TGGTGGTTTGCTACGACGT
**AP-2**	CATGGCCAAGCCCAACAT	CGCCCAGTTTGAAGGAAATC
**HMGB-1**	TAAGAAGCCGAGAGGCAAAA	AGGCCAGGATGTTCTCCTTT
**GAPDH**	TGTGTCCGTCGTGGATCTGA	TTGCTGTTGAAGTCGCAGGAG
